# Association of Vitamin D with the TaqI Polymorphism of the VDR Gene in Older Women Attending the Basic Health Unit of the Federal District, DF (Brazil)

**DOI:** 10.1155/2020/7145193

**Published:** 2020-09-24

**Authors:** Renata de Souza Freitas, Caroline Ferreira Fratelli, Calliandra Maria de Souza Silva, Luciano Ramos de Lima, Marina Morato Stival, Izabel Cristina Rodrigues da Silva, Silvana Schwerz Funghetto

**Affiliations:** ^1^Graduate Program in Health Sciences and Technologies, Faculty of Ceilandia, University of Brasilia, Federal District, Brasília, Brazil; ^2^Faculty of Ceilandia, University of Brasilia, Federal District, Brasília, Brazil

## Abstract

Aging is accompanied by various functional modifications determined by their environment, lifestyle, nutrition, and genetics. Based on these factors, it is essential to verify the vitamin deficiency in the elderly population. Hypovitaminosis D is commonly present in human aging, increasing the chances of developing noncommunicable chronic diseases. The VDR gene TaqI polymorphism may modify the vitamin D metabolic pathway by altering the interaction between the vitamin D receptor and the active circulating vitamin D. Therefore, this study aimed to investigate the association between serum vitamin D and biochemical and genetic factors, considering the TaqI polymorphism of the VDR gene, in an elderly population of the Federal District. The study was a descriptive, case-control, quantitative, and cross-sectional type and was conducted in two basic health units in the administrative region of Ceilândia, Federal District, DF, Brazil, with women aged 60 years or older. Anthropometric, biochemical, and genetic parameters (VDR TaqI polymorphism) were evaluated. The adopted significance level was 5%, and statistical analyses were performed using the SPSS version 20.0 program. The study consisted of 128 participants. The most prevalent age was from 60 to 65 years (*N* = 53; 41.4%). 66 elderly (51.6%) were part of the case group (hypovitaminosis D), while 62 were in the control group. In the case group, 30.2% had grade I obesity, 77.3% were hypertensive, and 51.5% were diabetic. The TT genotype was present in 47% of the case group and 54.8% in the control group (*p*=0.667). There was no association between serum vitamin D levels and the VDR gene variant TaqI polymorphism in an elderly Brazilian population.

## 1. Introduction

Population aging is a growing transformation in Brazil. This demographic transition may be elucidated (inferred) by changes in the mortality and the birth rates, as well as the populations' nutritional, economic, social, and disease profiles [1, 2].

According to the Brazilian Institute of Geography and Statistics (IBGE), there will be about 7 million more women than men by 2050 in Brazil, and the elderly will probably be represented by 76 older men per 100 older women [3].

Aging happens with increasing age, emerging structural, and functional changes in the body. The observed physiological changes increase over the years being discreet and natural. Nevertheless, such changes can lead to loss of function or disease. Biological changes may develop in various ways depending on the individual's genetics and lifestyle, influenced by psychological, cultural, and social aspects [4–6].

Vitamin D is a group made up of four cholesterol-derived molecules with a basic steroid structure and can, therefore, be classified as a steroid hormone. It participates in various processes in the human body, acting primarily in the regulation of calcium homeostasis, bone formation, and resorption and interacting as well with parathyroid, kidney, and intestine [7–9].

Vitamin D functions as a steroid hormone and is produced mainly in the skin exposed to ultraviolet radiation or absorbed in food. It participates in cellular processes and acts in the body's homeostasis, mainly in phosphocalcic metabolism. Its active form 25 (OH) D levels are considered deficient when below 20 ng/mL and insufficient when between 21 and 29 ng/mL [9–12].

For vitamin D to perform its various functions within the body, it needs to bind to the vitamin D receptor present in almost every cell in the body. This receptor is located in the cytoplasm in its free form. By binding to the active form of vitamin D, it translocates to the nucleus where it will associate with various genes, producing diverse biological effects [13–17].

Calcitriol acts through the vitamin D receptor. It is produced by the VDR gene (vitamin D receptor) and is a member of the nuclear receptor superfamily that regulates gene transcription (71). It is located on chromosome 12 (12q13.11) and has 12 exons [18].

Among the various allelic variants identified in the VDR gene are the TaqI and FokI polymorphisms, which are single nucleotide polymorphisms (SNPs). They can modify the vitamin D metabolic pathway by altering the interaction between the vitamin D receptor and active circulating vitamin D [19–22].

The TaqI polymorphism (rs731236) is located in exon 9 at the 3′-UTR end (nontranslated region) of the VDR gene. It results from a T (thymine) replacement by a C (cytosine) nucleotide, where the ATT codon transitions to ATC—generating a silent mutation, as both encode the amino acid isoleucine. Nonetheless, this SNP may change some functional characteristics of the protein [20, 23–25].

Thus, this study aims to investigate the association between serum vitamin D and biochemical and genetic factors, considering the TaqI polymorphism of the VDR gene in an elderly population of the Federal District.

## 2. Materials and Methods

### 2.1. Research Participants

This is a quantitative, descriptive case-control study (case: hypovitaminosis D; and control: vitamin D sufficiency) with cross-sectional design in older women attending a Basic Health Unit (BHU) in the Federal District (DF), Brazil.

The sample was selected from older women who volunteered to participate and were part of one of the Family Health Strategy (FHS) teams from two preselected BHUs.

In total, 128 participants were selected. The inclusion criteria were that all participants should be female, aged 60 years or over, and able to understand, verbalize, and answer the proposed questions. Contrarily, participants with mental illness or cancer undergoing treatment or who have had heart surgery in the last six months or who are taking vitamins or dietary supplements were excluded from the study.

The older women signed the Informed Consent Form (ICF) and a nursing appointment was scheduled, in which the identification form was completed with data such as weight and height as well as health and social-economic factors. Additionally, the participants signed the Biological Material Guard Term, and a venous blood collection was performed.

This study is approved by the Research Ethics Committee (CEP) of the Health Sciences Teaching and Research Foundation (FEPECS) of the Federal District State Department of Health (SES-DF) under the opinion number 1.355.211.

### 2.2. Genotype Analysis

After collection, the samples were sent to the laboratory for biochemical and genetic processing.

For genetic analysis, deoxyribonucleic acid (DNA) was extracted from blood collected using Invitek's Invisorb Spin Blood Minikit (250) (catalog# CA10-0005, lot# 1031100300) with an average concentration of 20 ng/*μ*L. TaqI polymorphism (rs721236) was genotyped (rs721236) using the polymerase chain reaction restriction on fragment length polymorphism (PCR-RFLP) based analysis. The primers used were sense 5′-CAG AGC ATG GAC AGG GAG CAA G -3 ′and antisense 5′-GCA ACT CCT CAT GGG CTG AGG TCT CA -3'.

Amplification was performed using the following thermocycling conditions: 95°C for 5 minutes (initial denaturation), followed by 35 cycles of denaturation at 94°C for 30 seconds, then by 65˚C for 30 seconds, and succeeded by 72°C for 30 seconds for oligonucleotide annealing. The final extension occurred at 72°C for 10 minutes.

The PCR product for rs731236 is a 740 bp fragment that amplified from the exon 9 regions of the VDR gene. After its enzymatic digestion with TaqI restriction enzyme (Jena, Germany), the TaqI polymorphism is cleaved into three bands of 290, 245, and 205 bp, defined as mutant t (C) allele, while the appearance of two 490 and 245 bp fragments indicates the presence of the ancestral allele T (T). Therefore, the TT genotypes were determined as 490 and 245 bp; Tt as 490, 290, 245, and 205 bp; and tt as 290, 245, and 205 bp with regard to the TaqI polymorphism.

### 2.3. Statistical Analysis

For statistical analysis, absolute and relative frequency distribution was applied for categorical variables and means with their standard deviation for continuous variables—with continuous data expressed as mean ± standard error (SE). Hardy–Weinberg equilibrium adherence to the genotypic frequency in controls was analyzed by the chi-square test with a degree of freedom. The genotypic and allelic frequencies of the case group (hypovitaminosis D) were compared to the control group by the chi-square test in recessive and dominant models. The association of clinical characteristics for each genotype was analyzed using the chi-square test and Fisher's exact test with a significance level of 5%.

Odds ratios (ORs) of allelic and genotypic frequencies were also calculated, with a confidence interval (CI) of 95%. The statistical program used was SPSS version 20.0 (SPSS Inc., Chicago, IL, USA).

## 3. Results

In this study, of the 128 elderly participants, aged 60 or over, the hypovitaminosis D prevalence was 51.6% (66 individuals): 29.7% with insufficiency and 21.9% with a deficiency for 25(OH)D. Accordingly, the case group sample size was 66 participants, and the control group was 62.

Most of the research participants were aged between 60 and 65 years (41.4%), followed by 66 to 70 years (28.9%), were married (45.3%), were from the Northeast region (68,8%), and declared themselves to be brown skin color (44.5%). Moreover, they had studied for 5 to 8 years (38.3%), are inactive in the labor market (43%), and have the income of one minimum wage or less (50.8%), and practiced religion is Catholicism (68.8%). Most declared that they performed physical activity (73.4%), regularly slept (50%), had insomnia (31.3%), did not smoke (89.8%), and did not use alcoholic beverages (95.3%) (Table 1).

Some older women had preexisting illnesses prior to the research. 78.1% had arterial hypertension, 53.9% had diabetes mellitus, 2.3% had fibromyalgia, and 12.5% had an inflammatory disease. A few had more than one disease (Table 2).

The genotypic distribution of the TaqI polymorphism was consistent with Hardy–Weinberg equilibrium between the group of participants with hypovitaminosis D (*p*=0.857) and the control group (*p*=0.393).  Table 3 describes the genotypic distributions of the VDR gene TaqI polymorphism associated with the case and control groups, in which there was no statistical difference (*p*=0.677). The homozygous ancestral genotype (TT) was more prevalent than the other genotypes, both in the hypovitaminosis D group (47.0%) and in the control group (54.8%). The ancestral allele (T) presented a more significant presence in both groups, with 68.8% in the case group and 72.6% in the control group.

Table 4 presents the description of life habit characteristics, according to the TaqI polymorphism of the hypovitaminosis D and control groups. 82.4% of the control group participants with the ancestral genotype (TT) practiced a physical activity compared to 64.5% in the case group. As for the older women who presented the mutant allele (Tt + tt), 74.3% exercised compared to 71.4% in the control group.

In the sleep variable, 58.8% of participants in the control group with the TT genotype had regular sleep patterns compared to 41.9% in the case group. Likewise, 48.6% of the participants with the Tt + tt genotype in the case group had regular sleep patterns compared to 50% in the control group. Of the participants who had the ancestral genotype (TT), 90.3% did not smoke compared to 94.1% in the control group, while of the participants with mutated allele (Tt + tt), 91.4% of the case group did not smoke compared to 82.1% of the control group. Furthermore, 100.0% of the hypovitaminosis D group with Tt + tt genotype did not use alcoholic beverages compared to 89.3% of the control group.

The clinical characteristics, systemic arterial hypertension (SAH), and diabetes mellitus (DM), correlated to the TaqI polymorphism, TT and Tt + tt genotypes, divided into groups, showed no statistical difference, as shown in Table 5. In the case group with the mutated allele (Tt + tt), 77.1% of participants had SAH compared to 82.1% of the control group. In the ancestral genotype (TT), 51.6% of the case group had diabetes, compared to 44.1% of the control group. For inflammatory diseases with ancestral genotype (TT), 83.9% of the participants had no disease, compared to 85.3% of the control group.

Table 6 presents older women divided into obese/overweight and nonobese women associated with the genotypes of the TaqI polymorphism, divided into case and control groups. Of the older women with hypovitaminosis D and mutated allele (t), 88.2% were obese or overweight, compared to 88.9% in the control group. Of the ones with the ancestral genotype (TT) and part of the case group, 75.9% were obese or overweight compared to 90.6% of the control group. None of these differences were significant.

Biochemical analyses such as measurement of total cholesterol and fractions, liver and kidney function markers, glucose, and glycated hemoglobin (HbA1c) were obtained from the research participants.

Table 7 assesses the difference in means of these biochemical parameters between the study groups in the different genotypes. With regard to the ancestral genotype (TT), the mean of glucose, HbA1c, triglycerides, HDL, aspartate transaminase (AST), alanine transaminase (ALT), creatinine, and urea was not significantly different. However, when observing the total cholesterol averages (184.86 mg/dL) in the case group in relation to the control group (211.68 mg/dL), the difference was statistically significant (*p*=0.012). In the case of LDL cholesterol, the case group presented an average of 110.55 mg/dL against 130.52 mg/dL in the control group, and this variation was also statistically significant (*p*=0.050).

As for the Tt + tt genotypes, the parameters of HDL (*p*=0.046) and creatinine (*p*=0.039) presented statistical differences. Nevertheless, there was no significant difference found between mutated genotypes and the other biochemical tests analyzed.

## 4. Discussion

This study investigated several determinants for serum vitamin D levels, as well as whether the VDR gene's TaqI polymorphism is associated with vitamin D deficiency and insufficiency. Hypovitaminosis D is a significant health problem, especially in the elderly population. This deficiency/insufficiency may be related to life habits, food, sports, and obesity, as well as the presence of other pathologies [13, 26].

The prevalence of serum vitamin D insufficiency and deficiency in this study was 29.7% and 21.9%, respectfully. The study of Laird et al. [27] corroborates our findings, and it was performed with 5,356 participants in Ireland, in which 53.4% were women with an average age of 62.9 years. 13.1% of the studied population had vitamin D deficiency, regardless of the season. In the observed group of women, 20.3% had a 25 (OH) D deficiency. This study also evidenced that women over 80 years old and who lived alone were more predisposed to a decrease in serum vitamin D.

An analysis of Australian citizens estimated that 31% had vitamin D deficiency and linked this deficiency to increasing age, female gender, obesity, and physical inactivity [28]. A 37.3% prevalence of vitamin D deficiency was also noted in the Mexican population over 60 years [29].

Another critical factor is the skin color of the study participants; despite not having found an association, the group that presented hypovitaminosis D was composed of 34.8% white, 47.0% brown, and 13.6% black.

However, other studies show that skin color was related to hypovitaminosis D. Lutsey et al. [30] analyzed 12,215 participants, of which 56% were women with an average age of 57. This study also associated ethnicity and race with 25(OH)D serum levels, as black participants had an average of 18.2 ng/mL, and white participants had an average of 25.6 ng/mL. The lower vitamin D cutaneous production in people with black skin could explain this hypovitaminosis D. Hypovitaminosis D also correlated, in this study, with younger ages, gender (female), activities practiced, and obesity.

Another study also noted a higher prevalence of low 25 (OH) D concentration in black participants [31]. Melamed et al. [32] showed that non-Hispanic blacks are more likely to have vitamin D deficiency.

Regarding preexisting pathologies, there was no significant difference between the groups with low vitamin D concentration and the control group. Nonetheless, in the literature, an association between hypertension and vitamin D deficiency has been described. Vimaleswaran et al. [33] conducted a Mendelian randomization study that showed that an increase in vitamin D concentration reduces the risk of hypertension. Furthermore, the study of Kienreich et al. [34] reinforces the theory that hypovitaminosis D is a risk factor for hypertension. On the other hand, Gao et al. [35] conducted a longitudinal study and found that low 25 (OH) D levels were associated with the risk of the onset of prediabetes and type 2 diabetes in the Chinese population.

The present study investigated the distribution of the VDR gene TaqI polymorphism in older women with hypovitaminosis D (case group) compared to those with vitamin D sufficiency (control group). However, no statistically significant difference was found when the polymorphism genotype and allele frequency were analyzed with regard to low 25 (OH) D serum concentrations.

The literature has shown correlations of these polymorphisms with several pathologies, such as osteoporosis, risk of fractures, diabetes, metabolic syndrome, Alzheimer's disease, asthma, multiple sclerosis, susceptibility to tuberculosis, and cancer [12, 24, 36–42]. The present study analyzed the presence of specific pathologies with the TaqI polymorphism frequency. The mutated genotype (Tt + tt) had a slightly higher frequency of hypertension (82.1%) and diabetes (71.4%) in the control group, whereas, in the ancestral genotype (TT), hypertensive (77.4%) and diabetic (51.6%) frequencies were higher in the case group. Nevertheless, no statistical difference was found between these pathologies and TaqI polymorphism.

A case-control study conducted in obese Egyptian women showed that the carriers of the VDR gene polymorphic alleles, ApaI (Aa + AA), FokI (Ff + ff), and TaqI (Tt + tt), had significantly lower vitamin D serum values than those with the ancestral genotype [37].

Selvaraj et al. [43] observed that the presence of polymorphisms could influence vitamin D receptor presence. The study noted that, with TaqI polymorphism, there is a tendency for higher receptor levels in patients with ancestral genotype (TT) in comparison with those that presented the mutant allele (Tt + tt).

Nogueira et al. [44], in Brazil, showed, with regard to the VDR gene TaqI polymorphism, a frequency of 51.25% of the ancestral genotype (TT) in burn patients, but no association was found with hospital mortality. Carvalho et al. [45] found 73.1% of the heterozygous (Tt) genotype in older women, and, likewise, no association with osteoporosis was detected. Borges et al. [46] analyzed patients with chronic periodontitis and had a frequency of 60% heterozygous genotype (Tt) and 53.3% ancestral homozygous (TT).

Associations between biochemical parameters and vitamin D serum levels have been established. Some studies have shown that increased biomarker levels are associated with hypovitaminosis D, such as creatinine and uric acid [32, 47]. Nonetheless, in this study, no relationship was observed between all the biochemical variables analyzed and the 25(OH)D dosage, in quantitative terms. Although there was an association between the aspartate transaminase (AST) mean dosage and hypovitaminosis D, when evaluating this hepatic marker's altered and normal value, there was no correlation. One study evaluated these parameters in primary biliary cirrhosis and found that the increase in transaminases, AST and ALT, is correlated with low vitamin D levels [43]. De Paula Scalioni et al. [48] demonstrated in their study that people with high AST and cholesterol levels are more prone to low circulating vitamin D.

## 5. Conclusion

This study verified the nonassociation between vitamin D serum levels and the VDR gene variant, TaqI polymorphism, in an elderly Brazilian population. It also examined whether the mutated genotype (Tt + tt) could affect vitamin D serum concentration. However, no statistical difference was detected between the polymorphism studied and vitamin D level.

It is important to emphasize that this study did not evaluate vitamin D receptor protein and other polymorphisms found in the VDR gene, such as BsmI and ApaI, as it is not the focus of the study. Hence, future researches should contemplate these aspects.

## Figures and Tables

**Table 1:** Sociodemographic characteristics of older women attended at a Basic Health Unit (*N* = 128). Ceilândia, Brasília, DF, 2018.

Variable	Description	N	%
Age range	60–65 years	53	41.4
	66–70 years	37	28.9
	71–75 years	23	18.0
	76–80 years	10	7.8
	≥81 years	5	3,9
Marital status	Single	18	14.1
	Married	58	45.3
	Divorced	14	10.9
	Widowed	36	28.1
	No reply	2	1.6
Nationality (place of birth)	Northeast	88	68.8
	Midwest	15	11.7
	Southeast	19	14.8
	South	3	2.3
	North	1	0.8
	No reply	2	1.6
Color	White	47	36.7
	Mestizo	57	44.5
	Black	17	13.3
	Others	3	2.3
	No reply	4	3.2
Schooling in years of study	0–4 years	48	37.5
	5–8 years	49	38.3
	≥10 years	31	24.2
Occupation	Active	21	16.4
	Retired	43	33.6
	Away/removed	1	0.8
	Inactive	55	43.0
	No reply	8	6.2
Monthly income in minimum wage	≤1 salary	65	50.8
	2 to 3 salaries	35	27.3
	4 to 5 salaries	6	4.7
	≥6 salaries	2	1.6
	Without income	3	2.3
	No reply	17	13.3
Religion	Catholic	88	68.8
	Evangelical	33	25.8
	Spiritist	2	1.6
	Atheist/agnostic	1	0.8
	Others	1	0.8
	No reply	3	2.4

*N*: number of participants; %: percentage; M: average; SD: standard deviation; min: minimum; max: maximum; ±: more or less; ≥: greater or equal; ≤: less than or equal; minimum wage: referent to the year 2017 in Brazil (nine hundred thirty-seven reais).

**Table 2:** Clinical characteristics of older women who attended a Basic Health Unit (*N* = 128). Ceilândia, Brasília, DF, 2018.

Variables		Groups				*p*	OR	CI
		25(OH)D insufficiency		Control				
		*N*	%	*N*	%			
Arterial hypertension	Yes	51	77.3	49	79.0	0.806^a^	0.902	(0.39–2.09)
	No	15	22.7	13	21.0			
	Total	66	100.0	62	100.0			
Diabetes mellitus	Yes	34	51.5	35	56.5	0.578^a^	0.819	(0.41–1.64)
	No	32	48.5	27	43.5			
	Total	66	100.0	62	100.0			
Fibromyalgia	Yes	2	3.0	1	1.6	0.999^b^	0.525	(0.05–5.93)
	No	64	97.0	61	98.4			
	Total	66	100.0	62	100.0			
Inflammatory diseases	Yes	10	15.2	6	9.7	0.348^a^	1.667	(0.57–4.90)
	No	56	84.8	56	90.3			
	Total	66	100.0	62	100.0			

a: chi-square test; b: Fisher's exact test.

**Table 3:** Genotypic and allelic distribution of the TaqI polymorphism associated with the hypovitaminosis D and control groups.

*TaqI*	Groups				*p*	OR	IC
	Hypovitaminosis D		Control				
	*N*	%	*N*	%			
TT	31	47.0	34	54.8	0.667	NA	NA
Tt	28	42.4	22	35.5			
Tt	7	10.6	6	9.7			
Total	66	100.0	62	100.0			
TT	31	47.0	34	54.8	0.374	0.729	(0.36–1.46)
Tt + tt	35	53.0	28	45.2			
Total	66	100.0	62	100.0			
T	42	31.8	34	27.4	0.442	1.235	(0.72–2.12)
T	90	68.2	90	72.6			
Total	132	100.0	124	100.0			

NA: not applicable; chi-square test.

**Table 4:** Distribution of life habit characteristics: physical activity, sleep, smoking, and alcoholism, according to the TaqI polymorphism of the hypovitaminosis D and control groups.

Variables	TT				*p*	Tt + tt				*p*
	Hypovitaminosis D		Control			Hypovitaminosis D		Control		
	*N*	%	*N*	%		*N*	%	*N*	%	
*Physical activity*										
Yes	20	64.5	28	82.4	0.202	26	74.3	20	71.4	0.267
No	10	32.3	6	17.6		9	25.7	6	21.4	
No reply	1	3.2	0	0		0	0	2	7.1	
*Sleep*										
Normal	13	41.9	20	58.8	0.340	17	48.6	14	50	0.454
Insomnia	11	35.5	11	32.4		12	34.3	6	21.4	
Difficulty sleeping	6	19.4	3	8.8		6	17.1	7	25	
No reply	1	3.2	0	0		0	0	1	3.6	
*Smoking*										
Yes	2	6.5	2	5.9	0.568	1	2.9	4	14.3	0.239
No	28	90.3	32	94.1		32	91.4	23	82.1	
No reply	1	3.2	0	0		2	5.7	1	3.6	
*Ethics*										
Yes	0	0	2	5.9	0.231	0	0	2	7.1	0.140
No	30	96.8	32	94.1		35	100	25	89.3	
No reply	1	3.2	0	0		0	1	3.6		

Pearson's chi-square test.

**Table 5:** Clinical characteristics associated with the TT and Tt + tt genotypes of the TaqI polymorphism in the case and control groups.

Variáveis variables	TT				*p*	Tt + tt				*p*
	Hypovitaminosis D		Control			Hypovitaminosis D		Control		
	*N*	%	*N*	%		*N*	%	*N*	%	
*Arterial hypertension*										
Yes	24	77.4	26	76.5	0.920^a^	27	77.1	23	82.1	0.624^a^
No	7	22.6	8	23.5		8	22.9	5	17.9	
*Diabetes mellitus*										
Yes	16	51.6	15	44.1	0.543^a^	18	51.4	20	71.4	0.107^a^
No	15	48.4	19	55.9		17	48.6	8	28.6	
*Fibromyalgia*										
Yes	2	6.5	1	2.9	0.602^b^	0	0	0	0	NA
No	29	93.5	33	97.1		35	100	28	100	
*Inflammatory diseases*										
Yes	5	16.1	5	14.7	0.999^b^	5	14.3	1	3.6	0.214^b^
No	26	83.9	29	85.3		30	85.7	27	96.4	

NA: not applicable; a: chi-square test; b: Fisher's exact test.

**Table 6:** Classification of obese/overweight and nonobese associated with the TT and TT + tt genotype, divided into case and control groups.

Variables	TT				*p*	Tt + tt				*p*
	Hypovitaminosis D		Control			Hypovitaminosis D		Control		
	*N*	%	*N*	%		*N*	%	*N*	%	
Obese/overweight	22	75.9	29	90.6	0.170	30	88.2	24	88.9	0.999
Not obese	7	24.1	3	9.4		4	11.8	3	11.1	

NA: not applicable; Fisher's exact test.

**Table 7:** Mean values of the biochemical parameters associated with TaqI polymorphism, divided into case and control groups.

Variables	TT										*p*
	Hypovitaminosis D					Control					
	*N*	Mean	SD	Min	Max	*N*	Mean	SD	Min	Max	
Glucose	31	125.4	60.9	56	322	34	107.8	41.4	62	243	0.175
HbA1c	31	6.6	1.6	4.7	10.8	34	6	1.2	4.6	9.5	0.090
Cholesterol	31	184.9	42.4	89	294.7	34	211.7	41.4	120	307	0.012
Triglycerides	31	138.1	62.6	52	270	34	168.5	79.9	62	441	0.095
HDL	31	48.2	13.2	23	85	34	48.1	6.7	36	60.3	0.969
LDL	31	110.5	39.7	35	210	34	130.5	40.6	33	234	0.049^*∗*^
AST	31	24.9	9.3	10	57	34	28.5	17.5	16	101	0.326
ALT	31	20.3	7.4	9	40	34	31.4	35.2	10	198	0.092
Creatinine	31	0.8	0.2	0.4	1.3	34	0.8	0.2	0.4	1.1	0.999
Urea	31	34.9	8.2	20	59	34	36	12.9	14	66	0.698

Variables	Tt + tt										*p*
	Hypovitaminosis D					Control					
	N	Mean	DP	Min	Max	N	Mean	DP	Min	Max	

Glucose	35	122.3	59.5	72	362	28	125	44.9	77	264	0.843
HbA1c	35	6.6	1.8	5.1	12.5	28	6.4	1.2	4.6	10	0.616
Cholesterol	35	200.5	47.3	121.1	281	28	198.2	49.8	127	351.4	0.852
Triglycerides	35	158.9	80.6	60	389	28	141.4	53.3	70	295	0.327
HDL	35	47.3	9	32.4	69.7	28	51.7	7.9	40	73.4	0.046^*∗*^
LDL	35	121.4	45.1	39	215	28	117	45.8	53	247	0.704
AST	35	25.5	10.9	9	59	36	24.9	8.6	13	46	0.797
ALT	35	26.5	13	9	63	36	22.4	9	11	54	0.126
Creatinine	35	0.7	0.2	0.4	1.3	36	0.8	0.2	0.5	1.3	0.039^*∗*^
Urea	35	30.7	12.6	10	60	36	35.1	13.4	11	76.2	0.159

Chi-square test; ^*∗*^*p* < 0.05.

## Data Availability

The patients' databank used to support the findings of this study are restricted by the Research Ethics Committee (CEP) of the Health Sciences Teaching and Research Foundation (FEPECS) of the Federal District State Department of Health (SES-DF) under the opinion number 1.355.211, in accordance with the Resolution 466/2012 of the Brazilian National Health Council (CNS), in order to protect the patient privacy. Data are available from Izabel Cristina Rodrigues da Silva, belbiomedica@gmail.com, for researchers who meet the criteria for access to confidential data.
